# The Effect of Quercetin Loading in Polylactic Acid-Based Electrospun Fibers on Their Antioxidant, Antibacterial and Antitumor Properties

**DOI:** 10.3390/molecules30112307

**Published:** 2025-05-24

**Authors:** Nikoleta Stoyanova, Tsvetozara Damyanova, Tsvetelina Paunova-Krasteva, Ani Georgieva, Reneta Toshkova, Mariya Spasova

**Affiliations:** 1 Laboratory of Bioactive Polymers, Institute of Polymers, Bulgarian Academy of Sciences, Acad. G. Bonchev Str., Bl. 103A, 1113 Sofia, Bulgaria; nstoyanova@polymer.bas.bg; 2 Centre of Competence “Sustainable Utilization of Bio-Resources and Waste of Medicinal and Aromatic Plants for Innovative Bioactive Products” (CoC BioResources), 1000 Sofia, Bulgaria; 3 Stephan Angeloff Institute of Microbiology, Bulgarian Academy of Sciences, Acad. G. Bonchev Str., Bl. 25, 1113 Sofia, Bulgaria; tsvetozaradamianova@gmail.com (T.D.); pauny@abv.bg (T.P.-K.); 4 Institute of Experimental Morphology, Pathology and Anthropology with Museum, Bulgarian Academy of Sciences, Acad. G. Bonchev Str., Bl. 25, 1113 Sofia, Bulgaria; georgieva_any@abv.bg (A.G.); reneta.toshkova@gmail.com (R.T.)

**Keywords:** electrospinning, quercetin, polylactic acid, water-soluble polymer, antioxidant, antibacterial and antitumor properties

## Abstract

Quercetin (QUE) is a bioactive flavonoid that is naturally present in various fruits and possesses many pharmacological activities. Despite its health benefits, the bioavailability of quercetin is relatively low due to its crystalline form and hydrophobic structure. An approach to overcoming these drawbacks is its incorporation into amorphous polymer matrices. PLA and PLA/PEG fibrous materials loaded with QUE were obtained by electrospinning. The XRD analysis revealed a visible decrease in the crystallinity of QUE after its incorporation into PLA and PLA/PEG fibers. The obtained fibrous materials and, especially, the PLA/PEG mat loaded with the flavonoid exhibited high antioxidant activity due to the better wettability and higher release rate of the bioactive compound. Moreover, the PLA/QUE and PLA/PEG/QUE mats possessed antibacterial properties against *Staphylococcus aureus* and *Pseudomonas aeruginosa*. Furthermore, the antitumor activity of the prepared mats was tested against SH-4 cancer cells and HaCaT keratinocytes. The obtained results reveal that the QUE-loaded fibrous mats exhibited high anticancer activity against cancer cells but lower toxicity to normal keratinocytes. The combined antioxidant, antibacterial and in vitro antitumor activities render these novel PLA-based materials loaded with QUE promising candidates for wound dressing applications and for application in local tumor treatment.

## 1. Introduction

Polyphenols represent a diverse and intricate class of naturally occurring compounds that are abundantly found in the plant kingdom. These compounds are characterized by the presence of multiple phenolic groups within their molecular structures, which contribute significantly to their unique chemical and biological properties [1]. Polyphenols have gained significant attention due to their potential health benefits, which are attributed to their antioxidant [2], anti-inflammatory [3] and antimicrobial [4] properties. Several studies suggest that polyphenols may help slow the progression of chronic diseases, such as diabetes [5], cancer [6] and cardiovascular conditions [7], by interfering with molecular pathways [8] critical to disease development. By neutralizing harmful free radicals and reducing inflammation, polyphenols help protect cells and tissues from damage, thereby lowering the risk of disease development [9]. Quercetin, a flavonoid and one of the most abundant polyphenols in the human diet, is particularly notable for its wide range of biological activities [10]. It is found in various foods, including onions, apples, and green tea [11]. Its capacity to modulate cellular processes, including those involved in apoptosis and cell cycle regulation, emphasizes its potential role in preventive and adjunctive therapies. [12]. Additionally, emerging research suggests a promising prospect of using quercetin to create innovative antimicrobial therapies, which is particularly relevant in the context of the growing concern over antibiotic-resistant bacteria, which poses a serious risk to public health and safety [13]. Quercetin has been shown to be effective against a variety of pathogens, including *Staphylococcus aureus* [14] and Escherichia coli [15], and may also enhance the activity of conventional antibiotics [16]. Furthermore, quercetin’s antibacterial activity is noteworthy, as it disrupts bacterial cell walls and inhibits bacterial enzymes, making it effective against a broad spectrum of bacterial infections [17]. One of the most promising areas of research involving quercetin is its anticancer activity [18]. Studies have shown that quercetin can induce cell death in cancer cells through multiple mechanisms, including the activation of caspases [19] and the modulation of signaling pathways involved in cell proliferation and survival [20]. Additionally, quercetin has been found to enhance the efficacy of chemotherapy drugs and reduce their side effects, making it a potential adjuvant in cancer treatment [21]. Research has demonstrated its efficacy against various types of cancer, including breast [22], colon [23], prostate [24], ovarian [25] and lung cancers [26], positioning quercetin as a promising candidate for cancer therapy due to its anti-angiogenesis [27], anti-metastatic [28] and antiproliferative properties [29]. Quercetin’s antioxidant activity is another key area of interest [30]. As a potent scavenger of free radicals [31], quercetin can protect cells from oxidative damage, which is implicated in the development of numerous diseases [32]. Beyond its antioxidant, anticancer, and antibacterial activities, quercetin also demonstrates potent anti-inflammatory effects by suppressing the production of inflammatory cytokines and enzymes and modulating the NLRP3 inflammasome [33], a key component of the immune response. This makes it beneficial in managing inflammatory conditions such as arthritis [34], as well as neuroinflammation [35].

The application of quercetin in electrospinning technology is a rapidly developing area of research. Electrospinning is a pivotal technique in nanotechnology, recognized for its cost-effectiveness and versatility in producing continuous polymer nanofibers on an industrial scale [36]. This process allows the fabrication of very fine nanofibers having diameters from 20 nm to several micrometers, facilitating diverse applications across medicine, pharmacy, industry, military systems, construction materials, electronics, agriculture and environmental remediation [37]. The incorporation of quercetin into electrospun fibers can enhance their bioactivity, making them suitable for various biomedical applications [38,39]. Studies have consistently demonstrated that quercetin-loaded electrospun fibers are capable of releasing the compound in a controlled manner [40], thereby providing sustained therapeutic effects [41]. This controlled release mechanism helps to sustain therapeutic concentrations of quercetin over time, which improves the compound’s overall efficacy. Moreover, the inclusion of polyphenolic compounds, such as quercetin, within electrospun fibers can leverage the unique properties of these fibers to optimize drug delivery. For instance, the use of a polymer blend system consisting of both hydrophilic and hydrophobic polymers has been shown to yield a promising release behavior, balancing the need for both the rapid and sustained release of quercetin [42]. In summary, quercetin, as a representative polyphenol, exhibits a myriad of health benefits, from anticancer and antibacterial activities to antioxidant properties. Its integration into advanced materials like electrospun fibers opens up new possibilities for its therapeutic applications. The PLA-PEG mechanical characteristics of electrospun fibrous materials might be tailored to meet the needs of certain tissues, expanding their use in wound healing, regenerative medicine and cancer treatment. Along with quercetin’s anti-inflammatory, anticancer, and antioxidant effects, these polymers’ synergistic actions provide a multipurpose platform that has great promise for solving challenging biological issues [43]. With its novel and efficient method of treating cancer and other illnesses, this advanced drug delivery technique promises to make advances in the field of biomedical applications.

## 2. Results and Discussion

In our recent study, it was found that electrospinning PLA and PLA/PEG fibers, which were obtained from a polymer solution with a concentration of 10 wt.%, resulted in the preparation of defect-free and homogeneous fibers. The obtained matrices based on PLA fibrous materials have shown the potential to be suitable carriers of biologically active compounds such as quercetin [43], *Melissa officinalis* extract [44] and *Portulaca oleracea* [45,46]. We have demonstrated that the loading of water-soluble polymer into the PLA matrix increases the quercetin solubility, wetting ability and release, and it shows anticancer activity against HeLa cells [43]. Based on the promising results obtained recently, further study concerning the antioxidant, antimicrobial and anticancer activity against a melanoma cancer cell line was performed. Moreover, a comparison of the material’s action on normal keratinocytes was accomplished as well.

### 2.1. Electrospinning of Fibrous Mats

Four distinct spinning solutions with varying compositions were electrospun for the current investigation. The dynamic viscosities of the spinning solutions were measured prior to electrospinning. The viscosity values recorded were 1700 cP, 1245 cP, 1885 cP and 1450 cP for PLA, PLA/PEG, PLA/QUE and PLA/PEG/QUE, respectively. The dynamic viscosity, as demonstrated by the values, is dependent on the presence of a low-molecular-weight polymer, in this case PEG, and the inclusion of the bioactive flavonoid quercetin. As can be observed, the addition of PEG to the spinning solution of PLA led to lower dynamic viscosity, so the low-molecular-weight polymer could be considered for a viscosity reducer. On the other hand, the viscosity values increased upon QUE inclusion in the PLA or PLA/PEG solutions. Investigating the composition/viscosity/fiber diameter connection requires measuring the spinning solution viscosities. Typically, the primary factor influencing the fiber diameter is thought to be the solution’s viscosity. In general, fiber diameters increase as concentration and solution viscosity increase.

**Figure 1:**
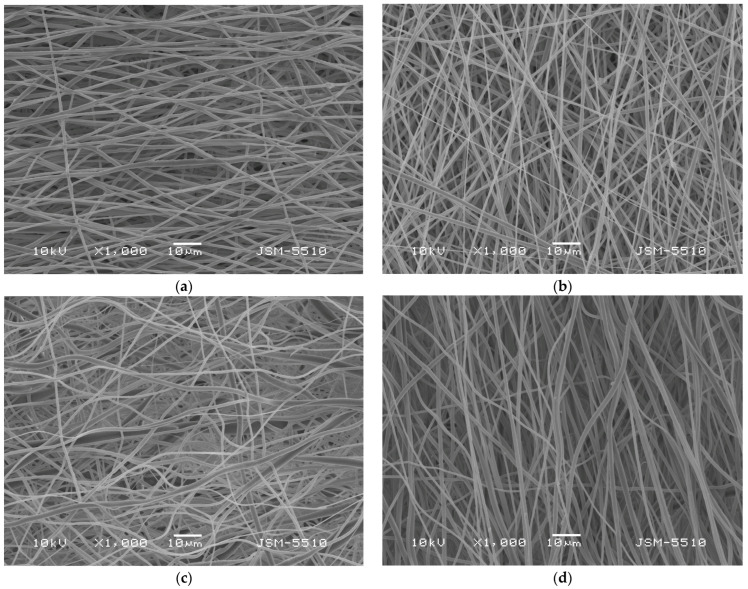
SEM micrographs of (**a**) PLA fibers, (**b**) PLA/PEG fibers, (**c**) PLA/QUE fibers and (**d**) PLA/PEG/QUE fibers. Magnification ×1000.

Figure 1 displays the morphology of the obtained fibrous materials, observed by scanning electron microscopy (SEM). SEM micrographs reveal that all four types of fibers were defect-free and cylindrical. ImageJ software (ImageJ 1.54 g) was used to analyze the SEM images in order to calculate the average fiber diameters. The production of PLA fibers with diameters of 768 ± 138 nm was achieved by electrospinning PLA solutions at a concentration of 10 wt%. Both the resulting fiber diameters and the dynamic viscosity of the blend solution were decreased by adding the second polymer, PEG with a lower molecular weight, to the PLA solution. It was found that the PLA/PEG fibers’ diameter had an average value of 671 ± 137 nm. The average fiber diameter of PLA and PLA/PEG fibers upon quercetin loading was insignificantly increased to 807 ± 159 nm and 713 ± 143 nm, respectively.

### 2.2. X-Ray Diffraction Characterization

**Figure 2:**
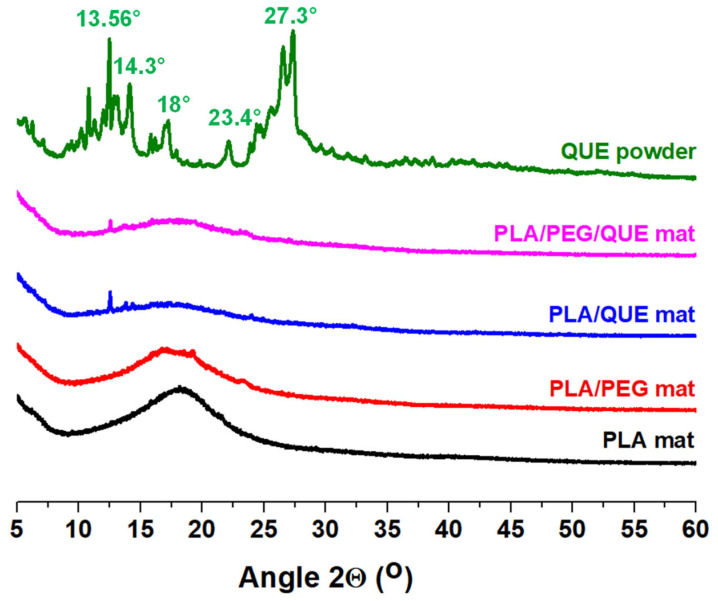
X-ray patterns of QUE (powder) and fibrous materials with and without QUE and PEG.

The X-ray diffraction examination of crystalline solids has been crucial in providing precise information on a variety of pharmaceutical properties, enabling the creation of more effective, alternative drugs and biologically active compounds. It is well known that amorphous pharmaceuticals have better dissolution and thus better bioavailability properties than their crystalline counterparts [47]. Figure 2 presents the XRD patterns of quercetin powder and the electrospun fibrous mats of PLA, PLA/PEG, PLA/QUE and PLA/PEG/QUE, analyzed within a 2θ range of 5° to 60°. The XRD pattern of QUE powder reveals sharp crystalline peaks, reflecting its highly ordered crystalline structure, with characteristic diffraction peaks observed at 2θ = 13.56°–14.3°, 15.7°–18.0°, 23.4° and 27.3°. When QUE is incorporated into PLA fibrous materials through electrospinning, a visible decrease in crystallinity is observed, indicating a transition toward an amorphous state (PLA/QUE mat). This amorphization is further enhanced by the addition of PEG, as demonstrated by the predominantly amorphous nature of the PLA/PEG fibrous material. In the PLA/PEG/QUE mat, the characteristic peaks of QUE are less intense, suggesting practically complete amorphization of this polyphenol compound. The obtained results reveal that, along with the applied electrospinning process, the presence of PEG facilitates the amorphization process, promoting an amorphous structure in the fibrous composites loaded with QUE.

### 2.3. Determining Antioxidant Activity

**Figure 3:**
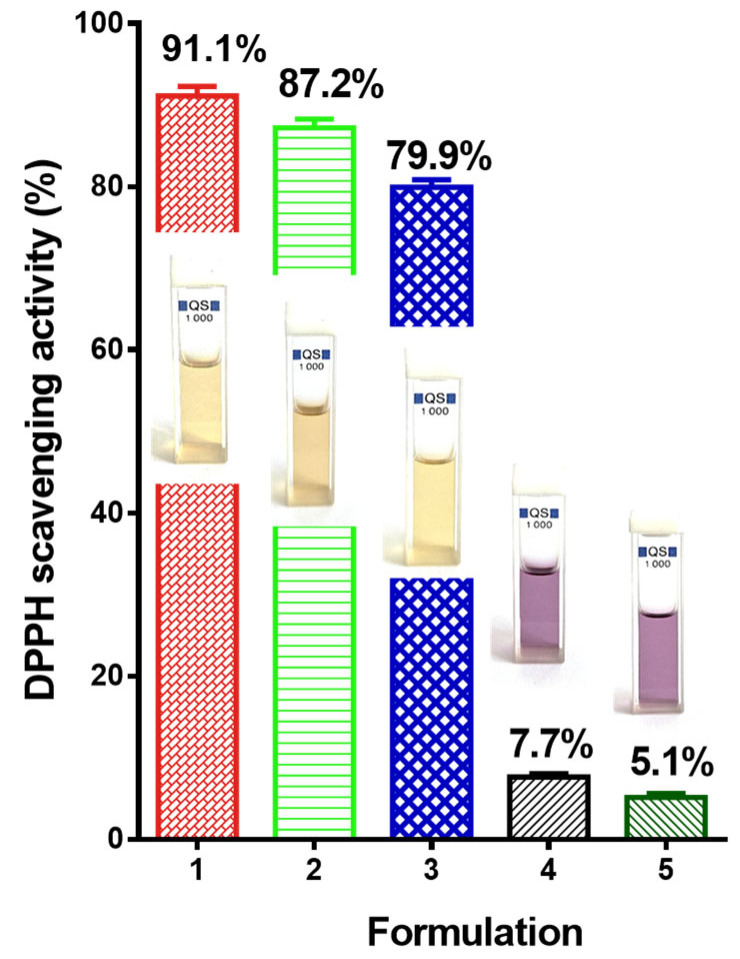
Antioxidant activity of 1—ethanolic solution of QUE, 2—PLA/PEG/QUE mat, 3—PLA/QUE mat; 4—PLA/PEG mat; and 5—PLA mat. All the samples were mixed with DPPH solution. Corresponding digital photographs of the studied solutions are presented as well.

Quercetin possesses strong antioxidant properties [31,48]. The antioxidant activity of PLA and PLA/PEG mats loaded with QUE was detected by using the DPPH radical scavenging assay. After 30 min of contact with the mats, the absorbance of a DPPH radical dot at 517 nm was determined spectrophotometrically (Figure S2). For the sake of comparison, the PLA and PLA/PEG mats were also tested. Figure 3 presents the antioxidant activity of all mats and the QUE solution. As can be seen, the PLA/PEG and PLA fibers do not show an impact on the DPPH solution. Their DPPH scavenging activity was 7.7% and 5.1%, respectively (Figure 3(4,5)). Moreover, the color of the DPPH solution does not change, and it remains violet. On the other hand, the mats loaded with QUE possess strong antioxidant activity (Figure 3(2,3)). Furthermore, the color of the DPPH solution after contact with the QUE-loaded mats changes to pale yellow. These results, as a value and the DPPH solutions’ color, are close to that of the QUE solution in ethanol. It is detected that the PLA/PEG-based fibers loaded with flavonoid showed a bit higher antioxidant activity in comparison to the mats based on PLA/QUE. This finding is most probably due to the better wettability and higher release rate of the bioactive compound [43]. All of these results show that when QUE was loaded into polyester-based fibers, its strong antioxidant capacity was maintained.

### 2.4. Antimicrobial Activity

Quercetin is a flavonoid known to compromise bacterial cell membranes, altering their permeability and leading to cell death. It also inhibits nucleic acid and protein synthesis, suppresses virulence factors, and inhibits biofilm formation [49,50,51,52].

**Figure 4:**
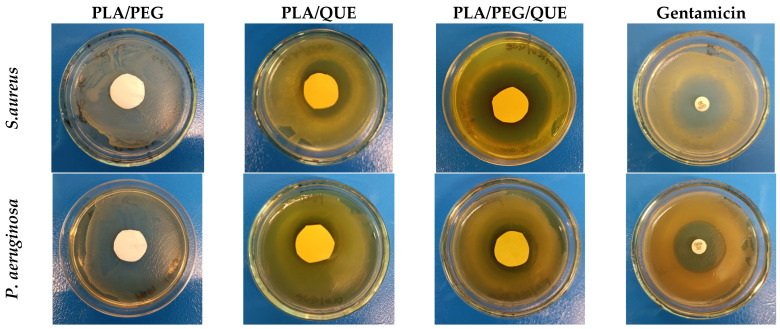
Disk diffusion test determining the antimicrobial activity of the tested electrospun fibers against *S. aureus* and *P. aeruginosa*. The results were obtained after cultivation with discs containing PLA/PEG, PLA/QUE and PLA/PEG/QUE mats. As a positive control, 30 µg/mL gentamicin discs were used.

**Figure 5:**
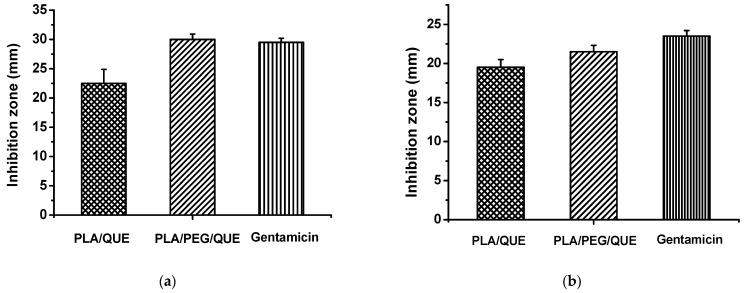
Evaluation of the inhibitory zone of the electrospun fibers measured in mm (mean ± SD), against (**a**) *S. aureus* and (**b**) *P. aeruginosa*.

To assess the antimicrobial efficacy of the obatined electrospun fibers, the inhibition at the zone of contact with the discs was evaluated. The bacterial strains used in this study are well-known causative agents of various infections, including chronic wounds, cystic fibrosis, chronic obstructive pulmonary disease, endocarditis, osteomyelitis and meningitis [53,54,55,56]. The presented figures illustrate the inhibition zones measured in millimeters (Figure 4 and Figure 5). The loaded discs with PLA/PEG/QUE, cultivated in the presence of *S. aureus*, showed the most distinct inhibition zones (Figure 4), ranging from 28 to 35 mm. In comparison, PLA/QUE discs also demonstrated growth suppression between 21 and 25 mm but with a significantly smaller inhibition zone, which still indicates a notable antimicrobial effect (Figure 5). Our data for *S. aureus* suggest excellent antimicrobial properties of quercetin encapsulated into the selected polymer fibers. The results for *P. aeruginosa* also showed higher inhibition for the triple combination of PLA/PEG/QUE, with a diameter zone from 23 to 24 mm. In contrast, the PLA/QUE test discs exhibited good antimicrobial efficacy, though with slightly lower inhibition zones ranging from 18 to 20 mm (Figure 4 and Figure 5). Furthermore, the antibacterial activity of blank PLA and PLA/PEG samples against *S. aureus* and *P. aeruginosa* was assessed. No inhibition zones were detected around the PLA and PLA/PEG fibrous mats (Figure S1 and Figure 4). When comparing the results for the two strains, we can conclude that the Gram-positive bacteria exhibit significantly higher sensitivity to the tested electrospun fibers loaded with QUE. Moreover, for both strains, the triple combination of PLA/PEG/QUE produced more pronounced effects, suggesting that the enhanced antibacterial activity is likely due to the faster release and diffusion of the encapsulated quercetin compared to the PLA/QUE combination.

Our findings correlate with other studies in the field, which have reported similar antimicrobial effects of quercetin against *S. aureus* and *P. aeruginosa* [57,58,59,60]. On looking in depth into the mechanisms of quercetin’s action, it has been shown to effectively suppress virulence factors in *S. aureus* by binding to casein hydrolase P (ClpP) and reducing its thermal stability. [61] demonstrated its significant role in inhibiting biofilm formation, pyocyanin production, protease and elastase activity in *P. aeruginosa* PAO1. The authors reported the downregulation of the genes lasI, lasR, rhII and rhIR as a result of quercetin’s action. These findings, viewed in the context of the growing incidence of untreatable bacterial infections caused by multidrug resistance, underscore the urgent need for new, effective antimicrobial agents. Considering the above, the present study proposes an innovative solution through the application of PLA-based electrospun fibers loaded with quercetin, demonstrating proven antimicrobial activity and potential inhibitory effects on bacterial virulence and pathogenicity, as a subject of future research and possible applications in medical practice.

### 2.5. In Vitro Cytotoxicity Tests

**Figure 6:**
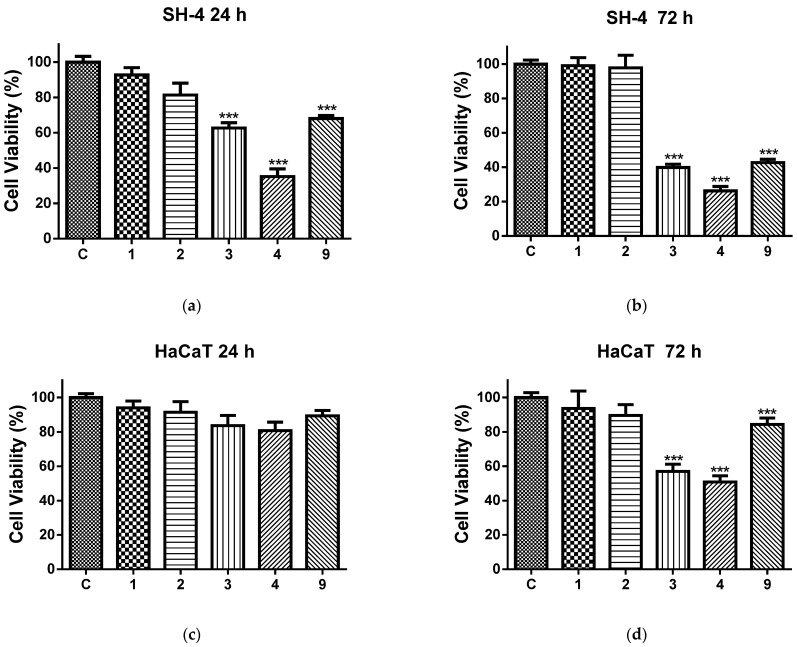
Cell viability of SH-4 cells (**a**,**b**) and HaCaT cells (**c**,**d**) evaluated by MTT assay after 24 h (**a**,**c**) and 72 h (**b**,**d**) contact with C—control (untreated cells); 1—PLA fibrous mat; 2—PLA/PEG fibrous mat; 3—PLA/QUE fibrous mat; 4—PLA/PEG/QUE fibrous mat; and QUE—free quercetin. *** *p* < 0.001.

The effect of the studied fibrous mats and free quercetin on the viability of melanoma cells and normal keratinocytes is presented in Figure 6. After 24 h of exposure to PLA and PLA/PEG mats, no statistically significant differences in viability between the treated and control SH-4 tumor cells were observed. In contrast, the viability of the melanoma cells cultivated in the presence of QUE-containing fibrous mats and free QUE was significantly reduced compared to the control (Figure 6a). The antiproliferative activity of the QUE-loaded electrospun mats was higher than that of the free QUE. The PLA/PEG/QUE mat was the most active among all tested samples and caused the strongest inhibition of cancer cell proliferation. With the extension of the exposure period to 72 h, the effects of the QUE-containing mats and the free QUE were further increased to values of 39.79% (PLA/QUE mat), 26.25% (PLA/PEG/QUE mat) and 42.71% (QUE), while the viability of the cells cultivated in the presence of PLA and PLA/PEG remained unaffected (Figure 6b).

We further analyzed the effect of the same fibrous mats on the viability of normal human keratinocytes (Figure 6c,d). The cultivation with the tested materials for 24 h did not induce a statistically significant reduction in cell viability (Figure 6c). PLA and PLA/PEG mats showed no cytotoxic effect, even after 72 h of treatment. The quercetin and QUE-containing fibrous mats applied for 72 h reduced the cell viability, and again, the decrease was highest in the cells treated with PLA/PEG/QUE mats (Figure 6d). However, the antiproliferative effects of the investigated materials on HaCaT cells were significantly less pronounced compared to those reported for the cancer cells.

### 2.6. Assay of Cell Death by Fluorescent Staining Methods

**Figure 7:**
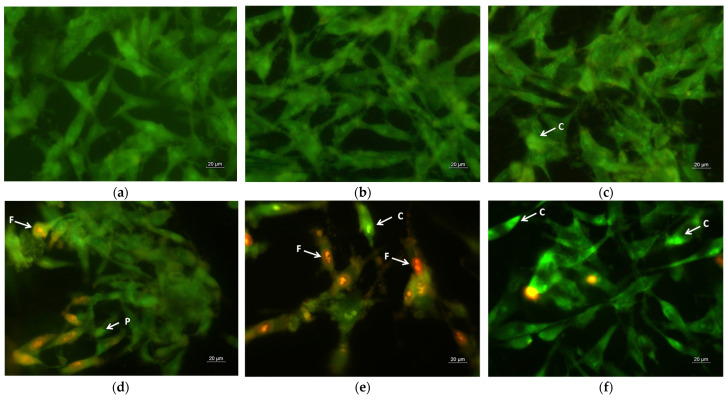
Fluorescent images of AO and EtBr double-stained SH-4 cancer cells incubated for 24 h with (**a**) untreated cells (control), (**b**) PLA fibers, (**c**) PLA/PEG fibers, (**d**) PLA/QUE fibers, (**e**) PLA/PEG/QUE fibers and (**f**) free QUE; C—chromatin condensation; F—nuclear fragmentation; P—pyknosis.

A hallmark of apoptosis is the alteration of cell and nuclear morphology, such as cell shrinkage, cell membrane bleb formation, cytoplasmic vacuolization, chromatin condensation, chromosomal DNA fragmentation, nuclear segmentation and the formation of apoptotic bodies. The apoptotic changes of the human SH-4 cells were visualized using dual staining with AO/EtBr, based on the differential uptake of the two fluorochromes (Figure 7). Acridine orange is permeable through intact cell membranes and stains the nuclei green, while ethidium bromide binds to DNA when membrane integrity is lost and marks the nuclei orange-red. The untreated (control) cells showed green fluorescence with homogeneous bright green nuclei and cytoplasm (Figure 7a). The morphology of SH-4 cells treated with PLA and PLA/PEG mats was similar to the control, with slight rarifying of the monolayer in the PLA/PEG mat (Figure 7b,c). Conversely, SH-4 cells incubated with quercetin-loaded fibrous mats (PLA/QUE and PLA/PEG/QUE) revealed early and late apoptotic cells with changes in cellular and nuclear morphology: distention, cytoplasmic vacuolization, blebbing of plasma membrane, chromatin condensation and DNA fragmentation and apoptotic bodies, in contrast with the controls (Figure 7d,e). The most severe changes were observed in melanoma cells after interaction with the PLA/PEG/QUE mat, where the monolayer confluence was highly reduced and the cells showed signs of late apoptosis (Figure 7e).

**Figure 8:**
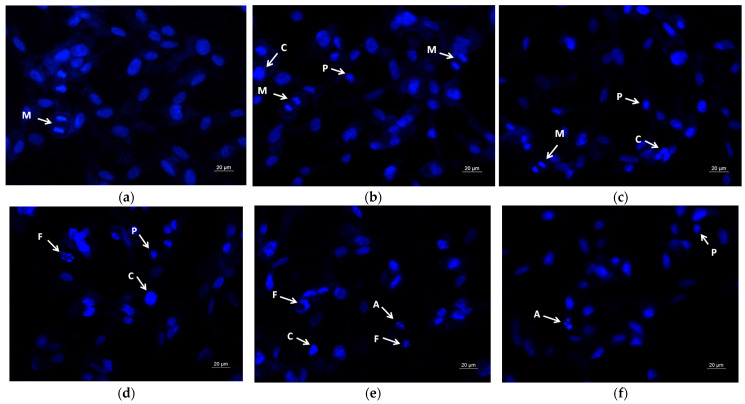
Fluorescent images of SH-4 cancer cells stained with DAPI: (**a**) untreated cells (control), (**b**) PLA fibers, (**c**) PLA/PEG fibers, (**d**) PLA/QUE fibers, (**e**) PLA/PEG/QUE fibers and (**f**) free QUE; A—apoptotic body; C—chromatin condensation; F—nuclear fragmentation; P—pyknosis; M—mitosis.

Further, DAPI staining was performed to evaluate the nuclear changes related to apoptotic cell death. As shown in Figure 8a, the control SH-4 cells exhibited intact round nuclei with evenly distributed and homogeneously colored chromatin. PLA treatment and PLA/PEG treatment did not influence the nucleus of the cancer cells, and morphological features similar to those of the controls were noted (Figure 8b,c). The SH-4 cancer cells treated for 24 h with QUE-loaded fibrous mats (PLA/QUE and PLA/PEG/QUE), as well as with QUE solution (100 μM/L), showed significant nuclear alterations, such as uneven nuclear contour, chromatin condensation, nucleus fragmentation, destruction of the nuclear envelope, and formation of apoptotic bodies (Figure 8d–f). The nuclei of PLA/PEG/QUE-treated cancer cells were the most severely damaged (Figure 8e).

As confirmed by AO/EtBr and DAPI staining (Figure 7 and Figure 8), our results clearly showed membrane and nuclear alterations in QUE-treated cells, indicating apoptotic cell death.

**Figure 9:**
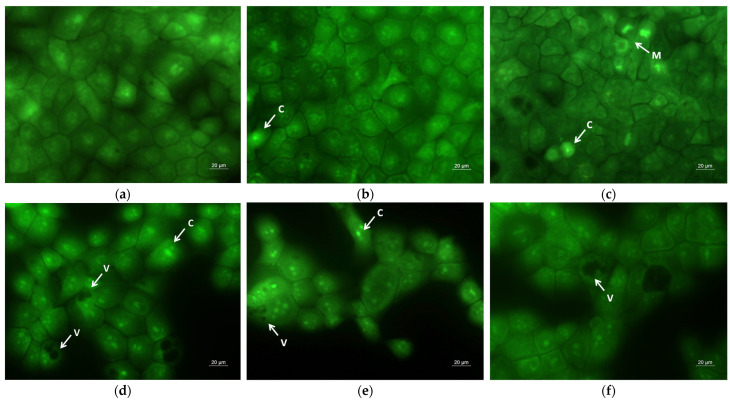
Fluorescent images of AO and EtBr double-stained HaCaT keratinocytes incubated for 24 h with (**a**) untreated cells (control), (**b**) PLA fluorescent fibers, (**c**) PLA/PEG fibers, (**d**) PLA/QUE fibers, (**e**) PLA/PEG/QUE fibers and (**f**) free QUE; C—chromatin condensation; M—mitosis; and V—cytoplasmic vacuolization.

**Figure 10:**
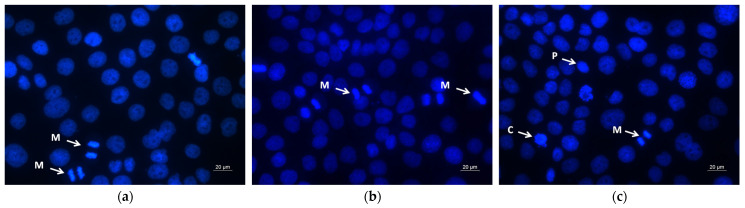

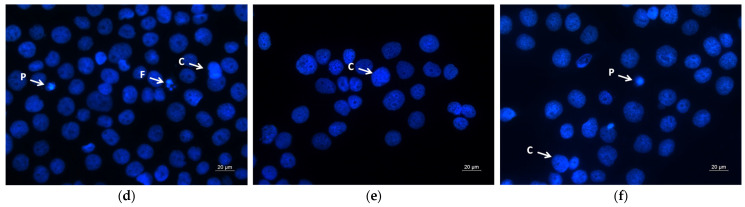
Fluorescent images of HaCaT keratinocytes stained with DAPI: (**a**) untreated cells (control), (**b**) PLA fibers, (**c**) PLA/PEG fibers, (**d**) PLA/QUE fibers, (**e**) PLA/PEG/QUE fibers and (**f**) free QUE; C—chromatin condensation; F—nuclear fragmentation; P—pyknosis; M—mitosis.

The morphology of HaCaT keratinocytes cultivated in the presence of the fibrous mats and free QUE was also examined by fluorescent staining with AO/EtBr (Figure 9) and DAPI (Figure 10). Both fluorescence staining methods showed that PLA and PLA/PEG fibers did not induce significant alterations in the HaCaT cell morphology (Figure 9b,c and Figure 10b,c).

After treatment with PLA/QUE and PLA/PEG/QUE fibers and free QUE, single cells with signs of early apoptosis, such as cell shrinkage, and intracytoplasmic vacuolization (Figure 9d–f), as well as pyknotic nuclei with condensed chromatin, were observed (Figure 10d–f). In the normal keratinocyte HaCaT cells, the effects of PLA/PEG/QUE fibers were the most clearly expressed (Figure 9e and Figure 10e). The obtained results are consistent with findings of the MTT assay and show the higher sensitivity of the SH-4 melanoma cancer cells in comparison with the normal keratinocytes.

## 3. Materials and Methods

### 3.1. Materials

A thermoplastic polyester-polylactic acid (PLA, Ingeo™ Biopolymer 4032D, NatureWorks, Minnetonka, MN, USA) with Mw = 259,000 g mol^−1^ and polydispersity = 1.94 and a water-soluble polyether-polyethylene glycol (PEG, Serva, Heidelberg, Germany) with molecular weight of 100,000 g mol^−1^ were used. The following solvents were used: dichloromethane (DCM; Sigma-Aldrich, Darmstadt, Germany), ethanol (abs. EtOH; Sigma-Aldrich, Darmstadt, Germany) and dimethyl sulfoxide (DMSO; AppliChem Darmstadt, Germany). They were of analytical-grade purity. The 2,2-diphenyl-1-picrylhydrazyl (DPPH) used in the study was purchased from Sigma-Aldrich (Darmstadt, Germany).

Fetal bovine serum (FBS) and Dulbecco’s Modified Eagle Medium (DMEM) were supplied from Gibco-Invitrogen (Leicestershire, UK). Penicillin–streptomycin solution was purchased from Lonza (Verviers, Belgium). Phosphate-buffered solution (PBS) and Trypsin–EDTA solution (2.5 g/L trypsin and 0.2 g/L EDTA) were supplied from AppliChem (Darmstadt, Germany). 3-[4,5-dimethylthiazol-2-yl]-2,3-diphenyl tetrazolium bromide (MTT) was delivered from Sigma-Aldrich Chemie GmbH (Darmstadt, Germany). Acridine Orange (AO) and ethidium bromide (EtBr) were supplied by Merck (Darmstadt, Germany).

### 3.2. Cell Lines and Culture Conditions

The human skin melanoma SH-4 (CRL-7724) obtained from the American Type Culture Collection (ATCC, USA ATCC, Rockville, MD, USA) and the non-tumorigenic cell line of human keratinocytes, HaCaT (CVCL_0038), obtained from the CLS Cell Lines Service (Eppelheim, Germany), were used in the present investigation. The cells were grown in Dulbecco’s Modified Eagle Medium (DMEM) supplemented with 10% heat-inactivated fetal bovine serum (FBS), 2 mM L-glutamine, 50 U/mL penicillin and 50 μg/mL streptomycin. Both cell lines were maintained in cell culture flasks at 37 °C in a humidified atmosphere with 5% carbon dioxide (CO_2_) and sub-cultured every 3–4 days to maintain exponential growth. Upon reaching confluency, the cells were trypsinized (0.25% trypsin-EDTA), counted and resuspended in fresh medium at the required concentration for each test.

### 3.3. Preparation of Fibrous Mats by Electrospinning

Prior to electrospinning, PLA, PLA/QUE, PLA/PEG and PLA/PEG/QUE solutions were prepared. Initial studies revealed that the optimal polymer concentration is 10 wt%. In the PLA/PEG mats, the ratio of PLA_80_/PEG_20_ *w*/*w* was used. The ratio of PLA to PEG was selected by preliminary experiments [62]. The QUE concentration was 10 wt% (to the polymer weight) [42,43]. The used solvent system contains dichloromethane/ethanol 80/20 *v*/*v*. For electrospinning, the prepared solutions were placed in a syringe (volume 5 mL) with a 20 G needle. The needle was connected to a high-voltage power supply working in the range from 10 kV to 30 kV. The applied high voltage was 25 kV. The distance between the tip of the needle and the drum was fixed at 12 cm. The fibers were collected on the drum, which was rotated at 1000 rpm. The spinning solutions were fed at a constant speed of 3 mL/h, ensured by an infusion pump (NE-300 Just InfusionTM Syringe Pump, New Era Pump Systems Inc., Farmingdale, NY, USA).

### 3.4. Characterization

#### 3.4.1. Dynamic Viscosity

The dynamic viscosity of the PLA, PLA/PEG, PLA/QUE and PLA/PEG/QUE solutions was measured with a Brookfield DV-II+ Pro (Middleboro, MA, USA) programmed viscometer fitted with a cone spindle CPE 52 for the one/plate option and a sample thermostatic cup at room temperature (25 °C).

#### 3.4.2. SEM Analysis

Scanning electron microscopy (SEM) was used to observe the structure of the prepared fibers. The SEM micrographs were captured using a JEOL JSM-5510 scanning electron microscope (JEOL Co. Ltd., Tokyo, Japan). Prior to examination, all samples were placed in a Jeol JFC-1200 fine coating machine and were gold-coated for 60 s under vacuum. At least thirty fibers from the SEM images were measured using ImageJ software, version 1.54 g, in order to determine the mean fiber diameter and the standard deviation.

#### 3.4.3. X-Ray Diffraction Analysis

X-ray diffraction (XRD) investigations were carried out at room temperature using a computer-controlled D8 Bruker Advance ECO powder diffractometer (Bruker, Billerica, MA, USA) with filtered Cu Kα radiation. With a step of 0.02° and a counting period of 1 s step-1, data were gathered in the 2θ range between 5° and 60°.

#### 3.4.4. DPPH Radical Scavenging Assay

The DPPH radical scavenging assay was used to examine quercetin’s capacity to scavenge free radicals. The capacity of the flavonoid QUE to donate hydrogen atoms was assessed by the decolorization of a 2,2-diphenyl-1-picrylhydrazyl (DPPH) ethanol solution. When antioxidants are present, the violet/purple color that DPPH creates in ethanol solution diminishes to yellow hues. The antioxidant activity of the prepared fibrous materials was determined. Ethanol solutions of QUE (5 × 10^−3^ M) or PLA, PLA/PEG, PLA/QUE and PLA/PEG/QUE fibrous samples (0.5 mg) were immersed in 3 mL of DPPH solution in ethanol (1 × 10^−4^ M). After incubation in the dark at room temperature for 30 min, the absorbance of each solution was measured at 517 nm by a DU 800 UV–VIS spectrophotometer (Beckman Coulter, Brea, CA, USA) in order to identify how many DPPH radicals were still present in the solution. The following equation was used to determine the antioxidant activity (AA%):

$$\mathrm{AA}\;\%=\left[\frac{\left(A_{DPPH}-A_{sample}\right)}{A_{DPPH}}\right]\;\times \;100\;$$

In the equation, the A_sample_—DPPH• is the solution absorption at 517 nm after the addition of the flavonoid solution or fibrous materials, and A_DPPH•_ is the absorption for DPPH• solution at 517 nm. All experiments were performed in triplicate.

### 3.5. Screening for Antimicrobial Activity—Disk Diffusion Assay

The antimicrobial effectiveness of the tested electrospun fibers was evaluated against two bacterial strains—*S. aureus* ATCC 29213 and *P. aeruginosa* ATCC 15692 [63], by performing a disk diffusion assay. To obtain the starting inoculum, the test cultures were incubated for 18 h at 37 °C in Tryptic Soy Broth (TSA, Sigma, Burlington, MA, USA). The resulting cultures were adjusted densitometrically according to the McFarland standard to a final cell concentration of 1 × 10^8^ CFU/mL, and 50 µL was spread onto Petri dishes. Discs with a diameter of 15 mm were sterilized by UV irradiation for 30 min on each side and placed onto the Petri dishes. To allow diffusion of the loaded samples from the discs into the agar, a pre-incubation was carried out for 2 h at 4 °C, followed by incubation for 24 h at 37 °C. Bacterial inhibition was assessed by observing a clear halo surrounding the discs. The size of this inhibition zone, measured in millimeters, indicated the level of antibacterial activity. For comparison, separate discs containing gentamicin (30 µg/mL) served as a positive control.

### 3.6. MTT Cytotoxicity Study

The MTT cytotoxicity test [64] was used to evaluate the impact of the various fibrous mats on the viability of SH-4 cells and HaCaT keratinocytes. In short, 0.25% Trypsin-EDTA was used to trypsinize the cells, and a hemocytometer was used to count them. A 96-well microtiter plate was used to transfer the cells at a concentration of 2 × 10^4^ cells/well. After an overnight incubation at 37 °C in a humid atmosphere with 5% CO_2_, the cells were placed in contact with different mats and free QUE. All the mats were preliminarily sterilized by UV light for 30 min. The incubation periods for the MTT test were 24 and 72 h. As controls, SH-4 cells and HaCaT cells were cultured only in medium or with QUE solution (100 μM/L). Five measurements were used to test each variant. After culturing with the fibrous mats, the SH-4 and HaCaT cells were washed twice with PBS and further incubated with 100 μL of MTT working solution (Sigma-Aldrich Chemie GmbH, Darmstadt, Germany) for three hours at 37 °C. The supernatants were then aspirated, and 100 μL of lysing solution (DMSO/ethanol 1:1) was added to each well to dissolve the formazan that resulted. An ELISA plate reader (TECAN, SunriseTM, Grodig/Salzburg, Austria) was used to perform the MTT test reading. The following formula was used to determine cell viability:

Cell viability (%) = OD (experimental)/OD (control) × 100/

### 3.7. Dual Acridine Orange/Ethidium Bromide (AO/EtBr) Fluorescent Staining

The morphological analysis of apoptosis was performed by double staining with acridine orange/ethidium bromide. The mixture of AO/EtBr was used to distinguish between live and dead cells based on the differential uptake of the two dyes. Viable and early apoptotic cells with intact membranes undergo penetration by AO, which fluoresces green. EtBr only emits orange-red fluorescence when it reaches cells with fractured membranes, such as dead and late apoptotic cells and necrotic cells. To obtain a cell monolayer, the cells were cultivated on sterile 13 mm diameter coverslips that were placed at the bottom of 24-well plates (2.0 × 10^5^ cells/well) for 24 h in a CO_2_ incubator. The following day, the QUE solution (at a concentration of 100 μM/L) and the analyzed electrospun materials were added to the cells. Negative controls were cells from the same line that were solely grown in medium. Following a 24 h incubation period, the coverslips were taken out and washed twice in PBS before being stained with the fluorescent dyes EtBr (5 µg/mL) and AO (5 µg/mL). Using a fluorescence microscope (Leica DM 5000B, Wetzlar, Germany), the morphological changes of freshly stained tumor and normal cells were studied and photographed within 10 min after staining to avoid fluorescence quenching.

### 3.8. DAPI—Fluorescent Staining

Nuclear morphology was examined by staining with the DNA-binding dye 4′,6-Diamidine-2′-phenylindole dihydrochloride (DAPI). As an effective agent for the visualization of nuclear DNA for both living and fixed cells, the DAPI molecule can penetrate an intact cytoplasmic membrane. As stated in the prior paragraph, the cells were cultivated on glass lamellae and exposed to different formulations. After 24 h of treatment, the cells were fixed with methanol and then incubated in the dark with DAPI solution (1 µg/mL in methanol) for 15 min at 37 °C. Mowiol^®^ was applied to stained cells on glass lamellae, and after coating, they were placed on slides and examined under a fluorescence microscope (Leica DM 5000B, Wetzlar, Germany).

### 3.9. Statistical Analysis

The statistical significance of the data was evaluated using the post hoc comparison test (Bonferroni) and one-way analysis of variance (ANOVA) in the GraphPAD PRISM program, version 5 (GraphPad program Inc., San Diego, CA, USA). Data are presented as mean ± standard deviation (SD), 95% confidence. For statistical significance, *p* < 0.05, ** *p* < 0.01 and *** *p* < 0.001 were used.

## 4. Conclusions

Morphological and structural analyses of PLA and PLA/PEG fibrous materials loaded with QUE by electrospinning were performed. The XRD study revealed that the flavonoid loaded into the fibers was amorphous, which is advantageous for use in medication dose formulations. The PLA/QUE and PLA/PEG/QUE fibrous materials possessed high antioxidant capacity, determined by the DPPH test. Furthermore, the QUE-containing mats were effective in inhibiting the growth of the Gram-positive bacteria *S. aureus* and Gram-negative bacteria *P. aeruginosa*. In addition, the QUE-loaded PLA-based materials manifested good anticancer activity against SH-4 cancer cells as a result of apoptotic induction. The new fibrous materials that were created show promise for use as wound dressings and for treating tumors locally.

## Data Availability

The data are contained within this article.

## Supplementary Materials

The following supporting information can be downloaded at: https://www.mdpi.com/article/10.3390/molecules30112307/s1, Figure S1. Disk diffusion test determining the antimicrobial activity of the tested PLA electrospun fibers against (a) *S. aureus* and (b) *P. aeruginosa.* Figure S2. UV/Vis spectrogram of reaction between the DPPH radical (red line) and PLA (yellow line), PLA/PEG (black line), PLA/QUE (blue line) and PLA/PEG/QUE (purple line) mats and QUE solution (green line).
