# Supplementary material for: The Effect of Quercetin Loading in Polylactic Acid-Based Electrospun Fibers on Their Antioxidant, Antibacterial and Antitumor Properties
Source: Molecules. 2025 May 24;30(11):2307. doi: 10.3390/molecules30112307 (PMC12155772; doi:10.3390/molecules30112307)
Supplement: Supplementary file 1 [file molecules-30-02307-s001.zip › molecules-3647114-supplementary.pdf]

Supplementary Material

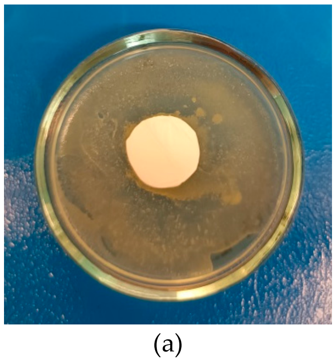
(a)

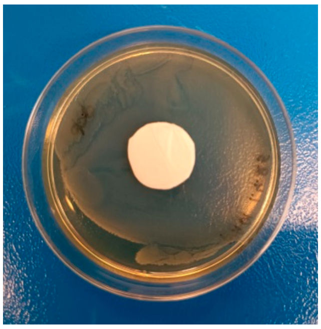
(b)

Figure S1. Disk diffusion test determining the antimicrobial activity of the tested PLA electrospun fibers against (a) *S. aureus* and (b) *P. aeruginosa.*

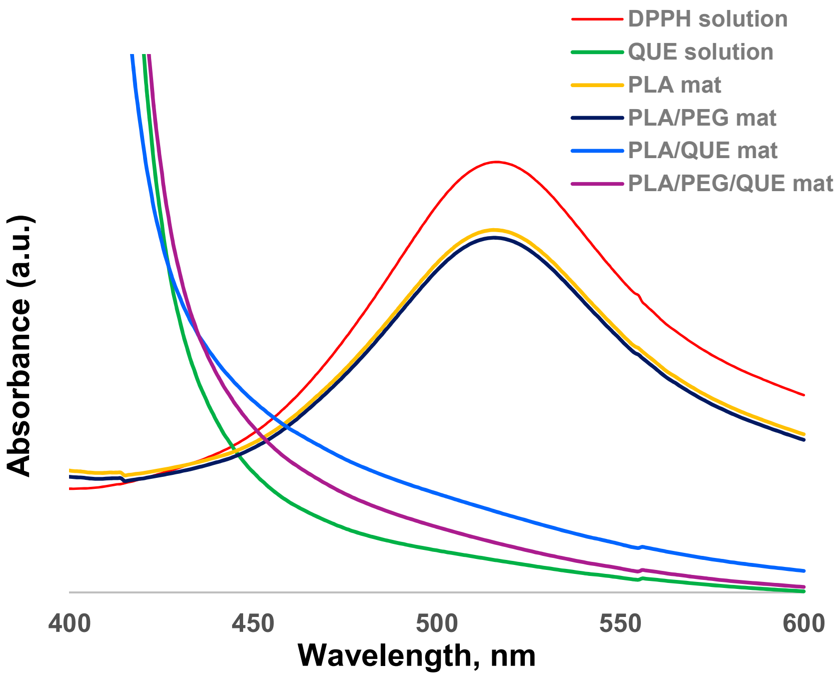

Figure S2. UV/Vis spectrogram of reaction between the DPPH radical (red line) and PLA (yellow line), PLA/PEG (black line), PLA/QUE (blue line) and PLA/PEG/QUE (purple line) mats and QUE solution (green line).
